# Comparison of Increase in Masticatory Efficiency Between Removable Partial Dentures Retained With Clasps and Retained With Attachments

**DOI:** 10.1002/cre2.70130

**Published:** 2025-04-22

**Authors:** Linda J. Dula, Kujtim Sh. Shala, David Stubljar, Andrej Starc, Shera Kosumi

**Affiliations:** ^1^ Department of Prosthetic Dentistry, Faculty of Medicine School of Dentistry Pristina Kosovo; ^2^ Department of Research and Development In‐Medico Metlika Slovenia; ^3^ Faculty of Health Sciences University of Ljubljana Ljubljana Slovenia; ^4^ Dental Faculty Alma Mater Europaea Campus College “Rezonanca” Prishtina Kosovo

**Keywords:** attachment, clasp, masticatory efficiency, removable partial dentures

## Abstract

**Objectives:**

This study aimed to evaluate and compare the masticatory efficiency of removable partial dentures (RPDs) retained with clasps versus those retained with attachments.

**Material and Methods:**

A total of 107 patients fitted with 138 RPDs participated in the study; 87 RPDs (63.0%) were clasp‐retained, and 51 RPDs (37.0%) were attachment‐retained. Subjects chewed 5.0 g of peanuts for 30 s, and masticatory efficiency was measured using a spectrophotometer at an absorption rate of 590 µm. Measurements were taken before insertion, immediately after, and 1 and 3 months post‐insertion of RPDs.

**Results:**

The analysis revealed progressive masticatory efficiency improvements for both clasp‐retained and attachment‐retained RPDs over time, with significant enhancements observed at the 3‐month post‐insertion mark. Initially, clasp‐retained RPDs showed slightly higher efficiency than attachment‐retained RPDs; however, attachment‐retained RPDs demonstrated superior efficiency after 3 months (*p* = 0.001). Consistent improvements were noted across different denture support types, with Triangular and Quadrangular supports showing the most notable gains by 3 months (*p* ≤ 0.006). GLMM analysis underscored the significant impact of time on masticatory efficiency (*F*(3, 511) = 4.926, *p* = 0.002), with no significant effects attributed to RPD type or support type alone, nor any significant interaction effects, indicating a universal improvement in masticatory function over time regardless of RPD design.

**Conclusions:**

RPD insertion significantly improves masticatory efficiency, particularly evident 3 months post‐insertion, with attachment‐retained RPDs outperforming clasp‐retained types. Improvements in masticatory function over time are consistent across all RPD designs, unaffected by denture type or support structure. This emphasizes the role of denture design in both immediate adaptation and long‐term treatment success, suggesting that time significantly contributes to enhanced masticatory efficiency regardless of RPD design, highlighting the importance of tailored prosthetic rehabilitation.

## 1 Introduction

Removable partial dentures (RPDs) are a form of prosthetic rehabilitation treatment that play an essential part in restoring patients' oral masticatory function, aesthetics, and phonetics. The request for this type of dentures is basically projected to grow due to an increase in human population and life longevity. RPDs with attachments were recommended over clasp‐retained RPDs due to higher patient satisfaction and improved oral health–related quality of life (OHRQL). These dentures provided better overall satisfaction and enhanced dental health outcomes (El‐Khamisy 2023).

To design useful RPDs, their biomechanic principles need to provide retention and stability support by focusing on the dispersion of forces into the supporting tissue (Yaparathna et al. 2024). Understanding the biomechanics also involves understanding of nature of surrounding tissue, namely oral tissue and teeth, to satisfy the right balance of prosthesis during its function. In other words, established RPDs are retained on the abutment natural teeth by clasps or attachments (Dosumu et al. 2005). However, sometimes the aesthetics might suffer; thus, the elimination of the buccal or labial direct retainer or clasp is crucial for establishing an aesthetically appealing look (Makkar et al. 2011). RPDs retained with attachment are more modern and fashionable in today's appearance‐oriented society than when they were first introduced. RPDs with attachments improve retention and stability of RPDs and also provide an aesthetic look, which when compared to the former metal display of the retentive clasp could not be achieved. The use of precision attachments in RPDs is regarded by some as the most advanced form of partial denture treatment. This method can enhance both the aesthetic and functional replacement of missing teeth and oral structures, often delivering superior results compared to traditional clasp‐retained RPDs (Khare et al. 2011; Oncescu Moraru et al. 2019). This rotation might always exist due to the mismatch of tissue resiliency and the abutment teeth, which have different viscoelastic reactions. The inclusive clasps transmit the greatest concentration of stress to the supporting structures in the photoelastic models (Costa et al. 2009). The use of ring clasps and precise attachments produce destructive forces on abutment teeth. Unfortunately, the use of resilient extra coronal attachment for distal extension RPD allows a limited degree of distal rotation to protect the natural abutment from torquing during function (Makkar et al. 2011).

Replacing missing teeth with RPDs is beneficial for patients as it provides a requisite for efficient chewing (Wallace et al. 2018). Fueki et al. (2016) showed that prosthetic restoration with RPDs profits masticatory efficiency (ME) in patients, who needed replacement of missing posterior teeth. Still, it was shown that ME was decreased by 40% in patients with missing teeth, and RPDs did not entirely compensate for this deficiency (Moore and McKenna 2016; Schimmel et al. 2017). Mixed research outcomes can be found on the establishment of ME after this prosthetic rehabilitation (Wöstmann et al. 2008). Nevertheless, ME is an important parameter for evaluating masticatory function (Felício et al. 2008). The ability to chew is not only an important function and sign of oral health but also an indicator of general health (Inukai et al. 2010). Furthermore, ME is frequently used as a measurement for the determination of an individual's ability to chew, crush, and/or grind food (Cicvaric et al. 2023). Numerous different clinical and laboratory methods have been developed to measure ME function such as fractional sieving, computer imaging (Elgestad Stjernfeldt et al. 2017), spectrophotometry (Elgestad Stjernfeldt et al. 2019), optical scanning, and measurement of the volume of masticatory muscle recorded by electromyography (EMG) (Goiato et al. 2007), and even can be evaluated by determining the mixing ability of a two‐colored chewing gum/wax or color‐changeable chewing gums (Halazonetis et al. 2013; Tarkowska et al. 2017; van der Bilt 2011). However, none of these methods stands out as preferable or better; therefore, the selection is difficult and complex as some of them have disadvantages in time consumption (Santos et al. 2006).

The aim of this study was to evaluate the short‐term changes in ME of patients who had received RPDs retained either with clasps or with precision attachments.

## 2 Materials and Methods

Overall, 107 patients with 138 RPDs participated in this study at the Prosthodontics Department of the University Dentistry Clinical Center in Pristina, Kosovo. The research proposal was approved by the Ethical Committee of the Faculty of Medicine, under approval number 1551. Written consent was obtained from all individuals participating in the study.

### 2.1 Patients' Selection

The inclusion criteria for patients were as follows: (i) individuals who had not previously worn an RPD but had undergone tooth extraction at least 3 months before the study; (ii) no presence of periodontal disease, history of diabetes, or temporomandibular joint disorders; (iii) residual ridges without excessive undercuts or redundant mucoperiosteum; (iv) jaw relationships classified as Angle Class I with sufficient inter‐arch space to accommodate RPD insertion.

All necessary dental treatments and restorations were completed before commencing RPD treatment. Patients demonstrated good oral hygiene and healthy periodontal tissues. They were advised and agreed to adhere to oral hygiene instructions, including mechanical teeth cleaning and denture maintenance, with the importance of oral hygiene reinforced at each visit.

The design of RPDs for each patient was based on the condition and positioning of the remaining teeth and the overall oral health status, ensuring that the mobility of abutment teeth did not exceed 1 mm. A total of 138 RPDs were analyzed, with some patients receiving more than one RPD. The distribution of the 138 RPDs was as follows: patients with natural dentition in the opposing jaw (*N* = 37); patients with fixed dentures in the opposing jaw (*N* = 22); patients with clasp‐retained RPDs in the opposing jaw (*N* = 23); patients rehabilitated with resin RPDs in the opposing jaw (*N* = 17); and patients with attachment‐retained RPDs in the opposing jaw (*N* = 8). Patients were divided into two groups: (1) those fitted with clasp‐retained RPDs using extra‐coronal direct and indirect retainers, and (2) those fitted with attachment‐retained RPDs. The allocation of patients to these groups was based on willingness to participate rather than randomization. Patient participation and adherence to RPD use were monitored throughout all phases of the study.

### 2.2 Designing RPDs and Prosthetic Procedures

The design of each subject's denture was informed by biomechanical factors, with a focus on the principles of retention, stability, and support. The support for the RPDs was categorized into linear, triangular, or quadrangular designs. Linear support was further subdivided into diametric, diagonal, and transversal types, as defined by Steffel (1962). The classification for partially edentulous patients was designated as Class I through Class IV, with the principal criterion being the system's ability to illustrate the relationship of dental support to edentulous areas (Phoenix et al. 2008).

The frame casts of the RPDs were constructed from cobalt–chrome–molybdenum (Co–Cr–Mo) alloys. Retention mechanisms included clasp‐retained extra‐coronal direct and indirect retainers, Ney clasps, and RPDs equipped with extra‐coronal precision attachments such as the Bar‐Dolder system. The latter remains a prevalent choice among patients, primarily for economic reasons.

The missing teeth were replaced with RPDs, each secured with two Ney clasps on the distal abutments on each side, in accordance with standardized clinical procedures. Depending on the specific denture support required, clasps were also utilized on indirect abutment teeth. For anterior teeth, Bonyhard clasps were employed. The fabrication and fitting process encompassed several key steps: preparation of abutment teeth to accommodate the RPD, taking impressions, fabricating and trying in the metal framework, employing the altered cast impression technique, trying in the teeth, and ultimately, delivering the RPD to the patient. Patients received comprehensive instructions on the correct use of their dentures. To mitigate secondary irritation potentially stemming from the RPD, which could affect the periodontium, patients were advised to return 24 h post‐fitting to address any issues related to sharp edges or pressure points. Moreover, stringent oral and denture hygiene practices were demonstrated to each patient, emphasizing the importance of maintaining both natural and prosthetic oral health.

For patients scheduled to receive RPDs alongside fixed dentures, the teeth were initially prepared for the bridge. Metal‐ceramic bridges were fabricated following standard protocols. Patients were provided with a surveyed bridge equipped with two extra‐coronal BAR attachments, as described by Dolder. This approach was chosen for economic reasons, as such attachments are not provided free of charge in our clinic. The bar was strategically placed in the pontic area relative to the crown and ridge, ensuring it did not interfere with the labial and lingual surfaces. This placement allowed for increased clearance between the bar and the tissue, with the bar positioned such that the path of withdrawal of the removable segment was divergent from forces exerted on the fixed portion.

Subsequently, new RPDs were constructed according to standardized clinical procedures. The definitive cementing of the fixed bridge and the delivery of the RPDs occurred upon the completion of the RPDs. Patients were advised to return after 24 h to address any issues. Additionally, patients received thorough instructions on proper denture use and hygiene practices.

### 2.3 ME Measurement

Groups 1 and 2 were evaluated to determine ME with their respective RPDs, based on the type of dental support. The measurement of ME is influenced by several factors, including the type, size, and shape of the food (1.5 cm) (Fan et al. 2009). A crucial aspect of this measurement is the recording of the number of chewing strokes during mastication. Peanuts were chosen for this study because of their relatively uniform size, which aids in standardization. Furthermore, peanuts do not require any special preparation before use. Each participant was provided with 5.0 g of peanuts, weighed on an electronic scale, and instructed to chew them for 30 s. Participants were asked to chew in a natural and bilateral manner, whereas the total number of chewing strokes within the 30‐s period was recorded at each evaluation time point.

At each assessment time point, the bolus and dentures were removed, rinsed, and then transferred into a 1000 mL measuring cylinder. Water was added to dilute the mixture until the total volume reached 1000 mL. This suspension was stirred for 1 min and allowed to settle for 2 min. Subsequently, one‐third of the suspension was carefully pipetted into spectrophotometry containers for analysis. The content of the cell was measured using LS‐722N‐2000 Spectrophotometer (Qingdao Pharmacypro Co. Ltd., Shanghai, China) at an absorption rate of 590 µm. These measurements were conducted and repeated 1 and 3 months following the insertion of the RPDs, adhering to the ME measurement methodology, as outlined by Fan et al. (2009).

### 2.4 Statistical Analysis

The statistical evaluation of our study was meticulously carried out utilizing SPSS software, version 21 (IBM, Armonk, NY, USA). Initial analyses focused on the demographics and clinical characteristics of the study population, as well as the categorization and distribution of the RPDs by support type and jaw among 107 patients. For the primary analysis of ME, descriptive statistics were employed in multiple contexts: to summarize the demographics and clinical characteristics of the study population; to delineate the distribution of RPDs, categorized by support type and jaw, across a sample of 138 RPDs; to record chewing strokes at various time intervals; and to detail ME at differing time points following the insertion of both clasp‐retained and attachment‐retained RPDs. Further stratification was applied to analyze ME at subsequent time points post‐insertion, categorized by denture support type for both clasp‐retained and attachment‐retained RPDs.

The normality of ME measurements at these various time points was rigorously assessed using both the Kolmogorov–Smirnov and Shapiro–Wilk tests. Given the observed deviations from normality, our analysis favored the use of Generalized Linear Mixed Models (GLMM) to explore ME over time. This choice was predicated on the GLMM's superior ability to manage data that deviate from normal distribution assumptions, thus accommodating the varying patterns of data distribution observed throughout the study's duration.

In addition to GLMM, the Independent‐Samples Kruskal–Wallis test was also utilized as part of our analytical arsenal to calculate test statistics and *p* values, further enriching our examination of the data. This non‐parametric test was especially valuable in instances where the data did not conform to the assumptions of normality, allowing us to assess differences across multiple groups without relying on these assumptions. The GLMM approach, alongside the Independent‐Samples Kruskal–Wallis test, enabled us to assess changes in ME over time, considering both fixed effects (e.g., type of RPD and time points) and random effects (e.g., individual variations among patients). This comprehensive statistical framework was critical for interpreting the nuanced impacts of clasp‐retained versus attachment‐retained RPDs on ME across different time intervals.

To further delineate the nuances of these changes, pairwise comparisons of ME over time were conducted, employing Bonferroni adjustments to account for the multiple comparisons issue, thereby maintaining the integrity of our statistical conclusions.

In all statistical tests, a significance level was set at *p* < 0.05, marking the threshold for statistically significant differences and associations within our data.

## 3 Results

A total of 107 patients wearing one or more RPDs were included in the study. The mean age of participants was 56.7 years (SD = 11.0). The gender distribution was 45.8% female and 54.2% male. Additional demographic details are presented in Table 1. The study examined 138 RPDs, of which 87 (63.0%) were clasp‐retained and 51 (37.0%) were attachment‐retained. Among these, maxillary RPDs accounted for 66 (47.8%) and mandibular RPDs for 72 (52.2%) (Table 2).

**Table 1:** Demographics and clinical characteristics of the study population.

Variable	Total (*N* = 107)
Age (mean ± SD, years)	56.7 ± 11.0
Gender, *n* (%)	
Female	49 (45.8)
Male	58 (54.2)
Residence and education, *n* (%)	
Urban residence	88 (82.2)
Secondary or higher education	86 (80.4)
Other diseases, *n* (%)	
Heart disease	18 (16.8)
Diabetes	10 (9.3)

**Table 2:** Categorization and distribution of RPDs (*n* = 138) by support type and jaw among 107 patients.

RPD types	Support type					Jaw	
	Linear Diametric	Linear Diagonal	Triangular	Quadrangular	Subtotal (%)	Maxilla	Mandible
Clasp‐retained RPDs	38 (57.6)	15 (68.2)	27 (69.2)	7 (63.6)	87 (63.0)	32 (48.5)	55 (76.4)
Attachment‐retained RPDs	28 (42.4)	7 (31.8)	12 (30.8)	4 (36.4)	51 (37.0)	34 (51.5)	17 (23.6)
Total	66 (100)	22 (100)	39 (100)	11 (100)	138 (100)	66 (100)	72 (100)
Total by Jaw						66 (47.8)	72 (52.2)

**Figure 1:**
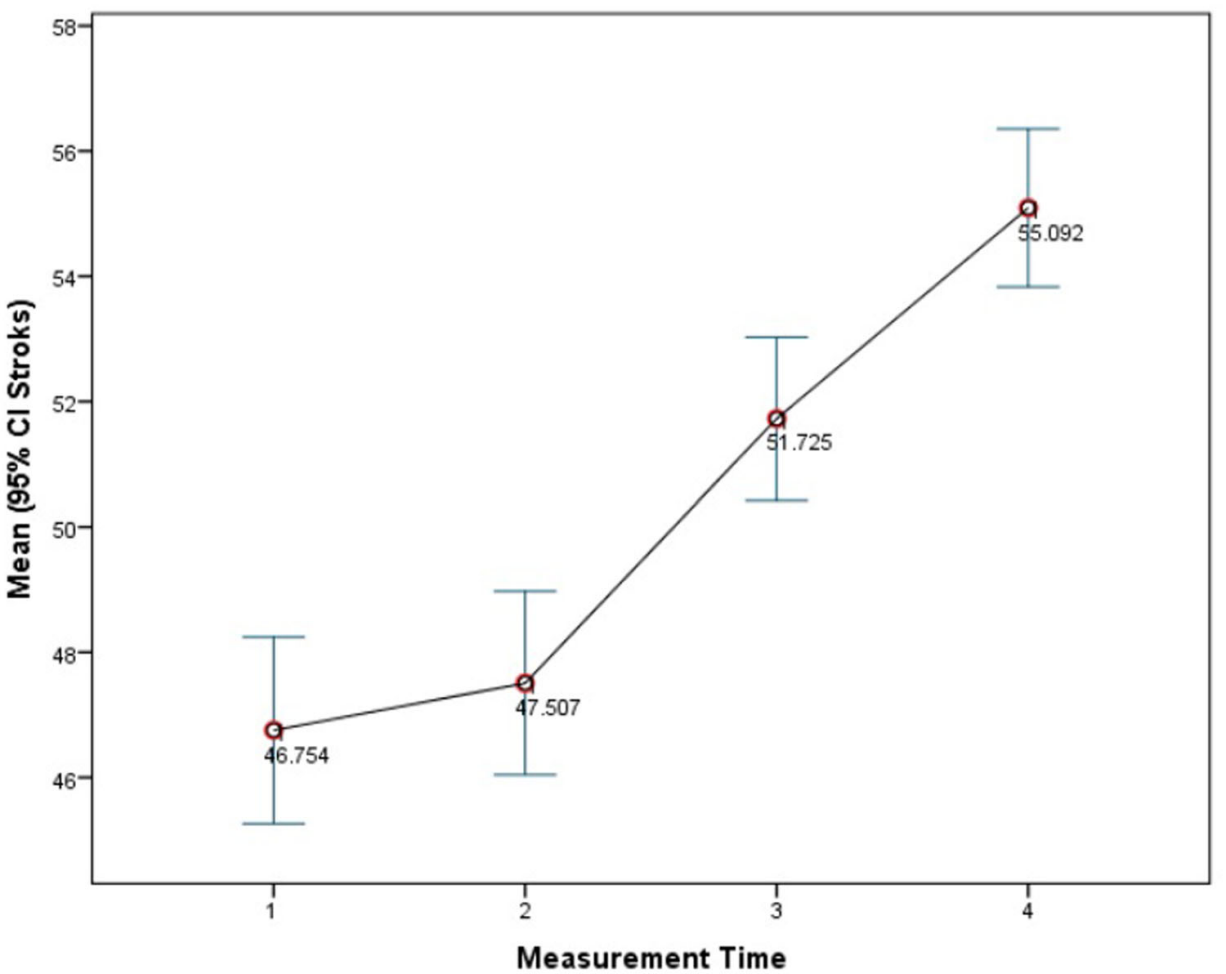
Descriptive statistics of chewing stroke measurements at different time points.

The average number of chewing strokes in patients before the insertion of RPDs was 46.8. This number slightly increased to 47.5 strokes immediately after the insertion of RPDs, further rising to 51.7 strokes 1 month post‐insertion and reaching 55.1 strokes 3 months after insertion. The data indicate a progressive increase in the number of chewing strokes from the time of RPD insertion, continuing through the 1 and 3‐month follow‐up periods (Figure 1).

The analysis of ME following the insertion of clasp‐retained and attachment‐retained RPDs demonstrates a progressive improvement over time for both types of RPDs. Initially, clasp‐retained RPDs showed a marginally higher mean efficiency (0.31) compared to attachment‐retained RPDs (0.27), a difference that was statistically significant (*p* = 0.02), indicating variability in baseline ME among patients.

Immediately after RPD insertion, the mean ME for both RPD types converged to 0.44, with no significant difference between the groups (*p* = 0.437). This suggests an immediate positive impact on masticatory performance from both RPD types. One month post‐insertion, both types of RPDs displayed further improvements, with attachment‐retained RPDs exhibiting a slightly higher mean efficiency (0.57) compared to clasp‐retained RPDs (0.55), although this difference was not statistically significant (*p* = 0.843), hinting at a comparable rate of adaptation or functionality between the two designs at this stage.

Three months after insertion, a significant divergence in efficiency was observed, with attachment‐retained RPDs achieving a higher mean efficiency (0.78) compared to clasp‐retained RPDs (0.72), a difference that was statistically significant (*p* = 0.001). This indicates a more pronounced long‐term improvement in ME with attachment‐retained RPDs.

**Figure 2:**
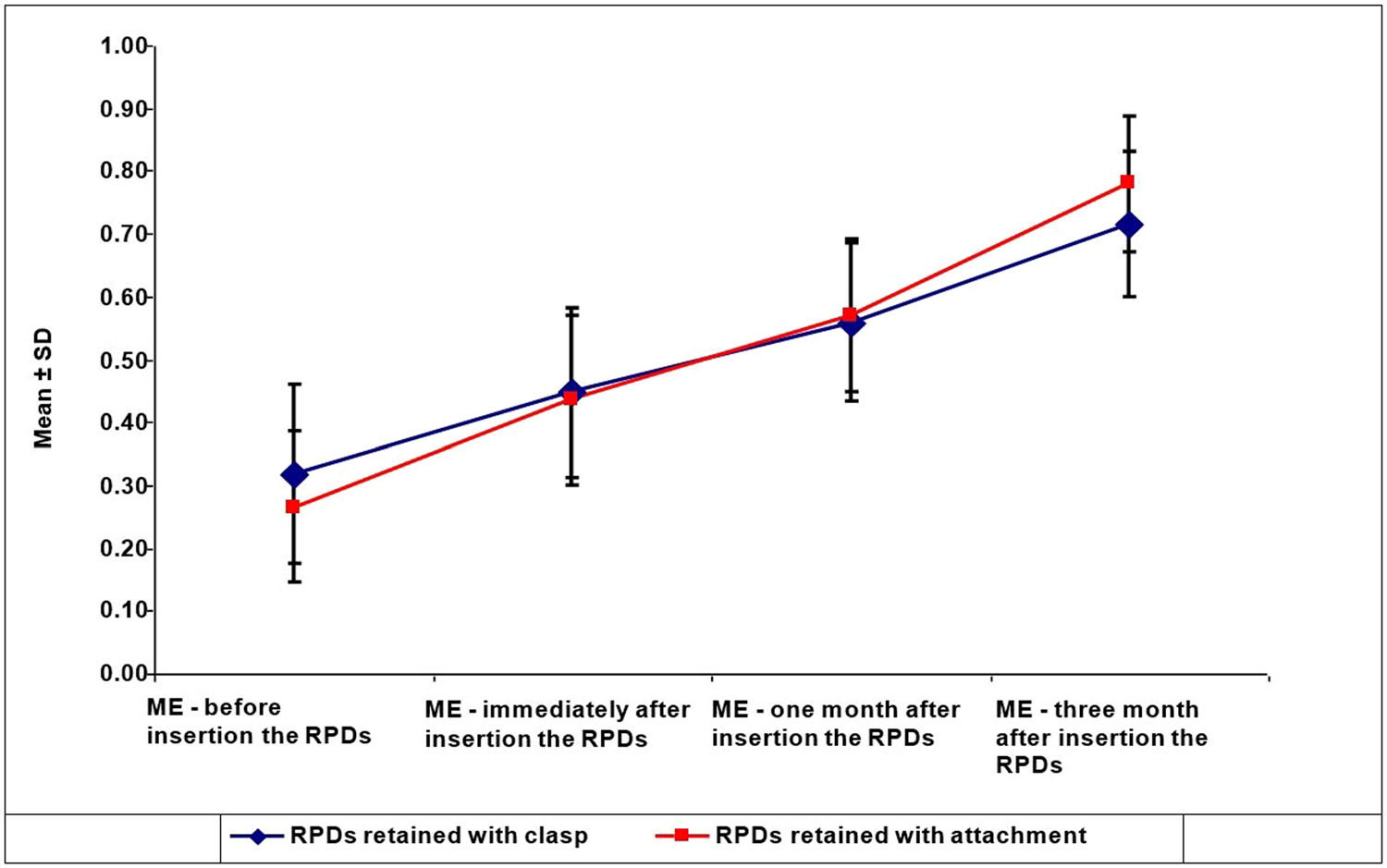
Changes in masticatory efficiency before, immediately after, and 1 and 3 months following the insertion of clasp‐retained and attachment‐retained RPDs.

These findings suggest that although both clasp‐retained and attachment‐retained RPDs lead to significant improvements in ME over time, the attachment‐retained RPDs might provide a slight edge in long‐term efficiency gains. The initial significant difference and the convergence of mean efficiencies immediately post‐insertion underscore the immediate benefits of RPDs. The subsequent significant improvement observed in attachment‐retained RPDs 3 months after insertion highlights their potential for a greater impact on masticatory function over time (Table 3 and Figure 2).

**Table 3:** Descriptive statistics of masticatory efficiency at different time points following insertion of clasp‐retained and attachment‐retained RPDs.

Time point and measurement	Clasp‐retained RPDs (*n* = 87)	Attachment‐retained RPDs (*n* = 51)	Test statistic^a^ and *p* value
Before RPD insertion			5.405, *p* = 0.020
Mean ± SD	0.31 ± 0.14	0.27 ± 0.12	
Range	0.04–0.66	0.05–0.56	
Immediately after insertion			0.603, *p* = 0.437
Mean ± SD	0.44 ± 0.14	0.44 ± 0.13	
Range	0.17–0.89	0.20–0.88	
1 month after insertion			0.039, *p* = 0.843
Mean ± SD	0.55 ± 0.13	0.57 ± 0.12	
Range	0.27–0.90	0.29–0.98	
3 months after insertion			11.496, *p* = 0.001
Mean ± SD	0.72 ± 0.12	0.78 ± 0.11	
Range	0.45–1.00	0.49–1.00	

*Note:* The normality of masticatory efficiency measurements was assessed using Kolmogorov–Smirnov and Shapiro–Wilk tests. Early deviations from a normal distribution were significant before and immediately after RPD insertion for both types, particularly for attachment‐retained RPDs (pre‐insertion *p* = 0.022, Shapiro–Wilk *p* = 0.007; post‐insertion *p* = 0.031, Shapiro–Wilk *p* = 0.039).

Analysis revealed specific denture support types demonstrating significant deviations from normal distribution patterns. Notably, the Linear Diametric group showed a substantial deviation before insertion (*p* < 0.0001), and the Linear Diagonal group immediately after insertion (*p* = 0.008).

^a^ The Independent‐Samples Kruskal–Wallis test was utilized to determine the differences between groups.

The analysis of ME across different denture support types within clasp‐retained RPDs reveals a consistent trend of efficiency improvement from before insertion through 3 months post‐insertion. Initially, mean efficiencies were closely aligned across the groups, with Linear Diagonal support showing a slight edge (mean efficiency of 0.34) over Linear Diametric (0.29), Triangular (0.33), and Quadrangular (0.30). Statistical analysis at this initial stage indicated no significant differences among the support types (*p* = 0.48), suggesting homogeneity in baseline ME.

After RPD insertion, all groups experienced an increase in mean ME, with the Triangular support achieving the highest mean value of 0.48. Although this post‐insertion improvement suggested an immediate benefit of RPDs, the statistical significance approached but did not reach conventional levels (*p* = 0.07), indicating a trend toward differentiation among the support types.

One month following insertion, the continuation of efficiency improvements was observed, especially in the Triangular and Linear Diagonal supports (mean efficiencies of 0.59 and 0.58, respectively). This period also suggested an increasing trend in efficiency, although the differences were not statistically significant (*p* = 0.109), highlighting continued patient adaptation across all denture support types.

By 3 months post‐insertion, all support types demonstrated significant increases in mean ME, notably with Triangular and Quadrangular supports achieving the highest values (0.75 and 0.77, respectively). This time point marked a statistically significant improvement in ME across the denture support types (*p* = 0.006), indicating substantial gains in function with prolonged use of RPDs.

Overall, these results affirm that clasp‐retained RPDs, regardless of the specific denture support type, significantly enhance ME over time. The most substantial improvements were observed 3 months after insertion, emphasizing the impact of denture design on optimizing masticatory function. The initial lack of significant differences evolves into marked efficiency gains, particularly evident 3 months post‐insertion, underscoring the importance of personalized denture design in enhancing oral rehabilitation outcomes (Table 4).

**Table 4:** Descriptive statistics of masticatory efficiency at different time points after insertion of clasp‐retained RPDs by denture support type.

Time point and measurement	Linear Diametric (*n* = 38)	Linear Diagonal (*n* = 15)	Triangular (*n* = 27)	Quadrangular (*n* = 7)	Test statistic^a^ and *p* value
Before insertion					2.48, *p* = 0.48
Mean ± SD	0.29 ± 0.14	0.34 ± 0.18	0.33 ± 0.15	0.30 ± 0.10	
Range	0.04–0.66	0.04–0.65	0.11–0.65	0.16–0.46	
Immediately after insertion					7.05, *p* = 0.07
Mean ± SD	0.40 ± 0.13	0.47 ± 0.18	0.48 ± 0.11	0.41 ± 0.10	
Range	0.17–0.71	0.17–0.89	0.18–0.67	0.22–0.51	
1 month after insertion					6.05, *p* = 0.109
Mean ± SD	0.50 ± 0.12	0.58 ± 0.16	0.59 ± 0.11	0.55 ± 0.07	
Range	0.32–0.82	0.33–0.90	0.27–0.75	0.43–0.61	
3 months after insertion					12.48, *p* = 0.006
Mean ± SD	0.66 ± 0.10	0.75 ± 0.12	0.75 ± 0.11	0.77 ± 0.12	
Range	0.45–0.92	0.60–1.00	0.52–0.93	0.64–0.99	

^a^ The Independent‐Samples Kruskal–Wallis test was utilized to determine the differences between groups.

The assessment of ME in attachment‐retained RPDs across various denture support types unveils a trajectory of improvement from insertion to 3 months post‐insertion. Initially, the Triangular support type exhibited the highest mean efficiency (0.33), whereas the Linear Diagonal support type presented the lowest (0.23), suggesting a variability in baseline efficiency among support types. The statistical analysis before insertion did not reveal significant differences across the groups (*p* = 0.092), indicating a comparable starting point for all support types in terms of ME. Progressing to immediately after insertion, all support types demonstrated an increase in mean ME, with the Triangular and Quadrangular support types showing marginally higher efficiencies (0.48 and 0.47, respectively). However, these early post‐insertion improvements did not reach statistical significance (*p* = 0.352), pointing to a uniform enhancement in masticatory performance across the board.

One month following insertion, the continued upward trend in ME was noted for all groups, with the mean efficiency reaching 0.60 for both the Triangular and Linear Diagonal supports. This phase of the study still did not show statistically significant differences among the support types (*p* = 0.292), underscoring a consistent improvement pattern across the board.

By the 3‐month mark, significant enhancements in mean ME were observed for all support types, with the Triangular support achieving the highest mean efficiency (0.80). Despite these observed improvements, the statistical test indicated no significant differences between the support types at this stage (*p* = 0.769), which may reflect the overall effectiveness of attachment‐retained RPDs in enhancing ME regardless of the specific support type.

Overall, the findings from Table 5 suggest that attachment‐retained RPDs facilitate progressive improvements in ME across all denture support types, with the most pronounced gains observed by the 3‐month follow‐up. The absence of statistically significant differences at each time point highlights the uniform benefit of attachment‐retained RPDs on masticatory performance, culminating in substantial efficiency enhancements, especially noted in the Triangular support group by the end of the study period.

**Table 5:** Descriptive statistics of masticatory efficiency at different time points after insertion of attachment‐retained RPDs by denture support type.

Time point and measurement	Linear Diametric (*n* = 28)	Linear Diagonal (*n* = 7)	Triangular (*n* = 12)	Quadrangular (*n* = 4)	Test statistic and *p* value
Before insertion					6.44, *p* = 0.092
Mean ± SD	0.24 ± 0.12	0.23 ± 0.11	0.33 ± 0.11	0.27 ± 0.12	
Range	0.07–0.56	0.05–0.36	0.19–0.52	0.18–0.45	
Immediately after insertion					3.27, *p* = 0.352
Mean ± SD	0.41 ± 0.12	0.45 ± 0.20	0.48 ± 0.12	0.47 ± 0.13	
Range	0.20–0.74	0.23–0.88	0.29–0.62	0.32–0.63	
1 month after insertion					3.73, *p* = 0.292
Mean ± SD	0.55 ± 0.11	0.60 ± 0.19	0.60 ± 0.09	0.60 ± 0.14	
Range	0.29–0.89	0.43–0.98	0.43–0.71	0.44–0.77	
3 months after insertion					1.13, *p* = 0.769
Mean ± SD	0.78 ± 0.11	0.76 ± 0.14	0.80 ± 0.10	0.78 ± 0.08	
Range	0.49–1.00	0.61–0.99	0.62–0.99	0.66–0.86	

The observed variations in normality underscore the use of GLMM for our analysis, providing flexibility to handle non‐normally distributed data and reveal nuanced patterns in ME over time and across RPD types.

The analysis was conducted to examine the main effects and interactions of type of RPDs, support types for RPDs, and time of measurements on the ME. The GLMM analysis indicated no significant ME for type of RPD (*F*(1.511) = 0.001, *p* = 0.973) or support type (*F*(3.511) = 0.089, *p* = 0.966), suggesting that neither the type of RPD nor the support type significantly influenced the ME on their own. However, there was a significant effect of Ttime (*F*(3.511) = 4.926, *p* = 0.002), indicating that the ME changed significantly over the different time points.

Regarding interactions, no significant interaction effects were found for type of RPD*support (*F*(3, 511) = 0.035, *p* = 0.991), type of RPD*time (*F*(3, 511) = 0.079, *p* = 0.971), support*time (*F*(9, 511) = 0.01, *p* = 1.0), or the three‐way interaction type of RPD*support*time (*F*(9, 511) = 0.07, *p* = 1.0). These results suggest that the effect of ME is not differentially influenced by combinations of RPD type and support type at the various time points measured (Table 6).

**Table 6:** Generalized Linear Mixed Model results for masticatory efficiency.

Source	*F*	df1	df2	Sig.
Corrected model	0.519	31	511	0.986
Type RPDs	0.001	1	511	0.973
Support type for RPDs	0.089	3	511	0.966
Time	4.926	3	511	0.002
TypeRPD*Support	0.035	3	511	0.991
TypeRPD*Time	0.079	3	511	0.971
Support*Time	0.01	9	511	1.0
TypeRPD*Support*Time	0.07	9	511	1.0

*Note:* The degrees of freedom method used was Satterthwaite's approximation. The significance level was set at 0.05, and *p* values were adjusted for multiple comparisons using the Bonferroni correction.

## 4 Discussion

RPDs continue to be perceived as the treatment of choice for missing teeth, especially in patients who have anatomical or financial restrictions on dental implant placement (Awawdeh et al. 2024). The degree of ME in patients with RPDs depends on their compromised support, retention, and stability, which are the most frequent issues associated with RPDs (Alqutaibi 2020). The number of patients requiring RPD treatment has increased through the last years (Schwendicke et al. 2020; Elagra et al. 2019). RPDs retained with attachment have an extraordinary function improving the aesthetic, functional, phonetic appearance, and comfort of the patient. They are mostly indicated in the shortened arch of the teeth and nonparallel abutment teeth (Jan et al. 2012). This intra‐individual study was conducted to compare the ME of clasps‐retained RPDs with extra coronal attachment‐retained RPDs.

Our study meticulously documented the progression of ME in patients fitted with both clasp‐retained and attachment‐retained RPDs, delineated by denture support types. The investigation encompassed 138 RPDs, offering a granular view of masticatory function evolution over a 3‐month period post‐insertion. For clasp‐retained RPDs, descriptive statistics unveiled a consistent trend of ME improvement across all denture support types (Linear Diametric, Linear Diagonal, Triangular, and Quadrangular) from the baseline to 3 months post‐insertion. Initial efficiencies were modest, with slight variances among the groups: the Linear Diagonal supports demonstrated a marginally higher mean efficiency before insertion. Notably, each subsequent time point—immediately after, 1 month, and 3 months post‐insertion—marked appreciable increases in mean ME for all groups. The Triangular and Quadrangular supports, in particular, showed remarkable enhancements, achieving the highest mean efficiencies 3 months after RPD insertion. These results suggest a positive impact of continuous RPD use on masticatory performance, with the greatest improvements materializing by the third month.

Similarly, attachment‐retained RPDs exhibited progressive improvement in ME across all denture support types from the point of insertion through to the 3‐month evaluation. Initially, the Triangular support group stood out with the highest mean efficiency. This upward trajectory of efficiency continued across all groups, culminating in substantial gains by the 3‐month follow‐up. The most pronounced enhancements were observed in the Triangular support group, closely followed by the other types, underscoring the effectiveness of attachment‐retained RPDs in bolstering masticatory function over time. These nuanced insights into the ME dynamics post‐RPD insertion highlight the transformative potential of both clasp‐retained and attachment‐retained RPDs. The differential enhancements observed across various denture support types further emphasize the importance of personalized denture design in optimizing oraln rehabilitation outcomes. Ultimately, our findings illuminate the substantial role of RPDs in improving ME, with the attachment‐retained RPDs, particularly those with triangular support, showing notable efficacy in enhancing masticatory function over the assessed period.

Similar was observed by Kapur (1991) who tested a total of 232 patients, including RPDs for 118 patients and fixed partial dentures (FPDs) for 114 patients. They tested patients at 16 weeks following RPD and thereafter at 6, 18, 36, and 60 months. In the beginning, there were no significant differences in ME and chewing strokes between the RPD and FPD groups. After treatment, FPDs and RPDs significantly improved ME of patients. The improvements in ME were slightly better in the RPD group than in the FPD group. After 60 months, only slight changes in ME performance were observed for both groups. In our case, longer time frame than 3 months was not tested, so it is difficult to discuss whether ME might improve even further. Furthermore, ME 3 months post‐insertion was higher in patients with attachment‐retained RPDs. A proper selection of RPDs plays a crucial role. The attachment is crucial for the success of distal extension RPDs (Nikolopoulou et al. 2019; Mousa et al. 2017). In studies by Persic et al. (2017) and Alqutaibi (2020), patient satisfaction among patients who received attachment‐retained RPD was found as higher compared to those who received conventional clasp RPD. The reasons for higher satisfaction were due to aesthetic outcome, stability while chewing, comfort, and speaking ability. In the current study, patient satisfaction was not tested, but another performance parameter such as the number of chewing strokes within 30‐s interval was measured.

The number of chewing strokes increased from the moment of RPD insertion at all measured intervals. The average number of strokes in patients before insertion of RPDs was 46.8 strokes, after insertion of RPDs was 47.5 strokes, 1 month after insertion of RPDs was 51.7 strokes and 3 months after insertion of the RPDs was 55.1 strokes. So ME of RPDs wearers improved with an increased number of strokes within a 30‐s interval. In general, patients with natural dentition had the highest ME (Toman et al. 2012; Berretin‐Felix et al. 2009). Several studies concluded that denture wearers on average need 4–8 times the number of strokes to achieve the same degree of food pulverization, depending also on the consistency of the food (van der Bilt 2011; Fuentes et al. 2018).

Despite all that, only minor differences were found in our study based on the type and design of RPDs with clasp or with attachment. ME was statistically significant and improved through time also for clasp‐retained RPDs. In these patients, with clasp‐retained RPDs, triangular and quadrangular denture support was better 3‐month post‐insertion when compared to the linear setting. In contrast, ME was highest in patients with attachment‐retained RPDs with triangular denture support, but there was no significant difference between the designs. This could not be proven due to the relatively small number of patients with triangular and quadrangular denture support of RPD. The literature suggests that ME with mandibular distal extension attachment‐retained RPDs can be improved when distal support is obtained from two distal implants strategically placed to counteract the inherent rotational movement during functional loading (Gonçalves et al. 2014; Putra Wigianto et al. 2021). However, among various factors on the degree of improvement in ME by wearing RPDs, bilateral missing posterior teeth might be a predictor for achieving clinical enhancement (Kikuchi et al. 2021). As discovered, ME performance was in agreement with studies conducted by Persic et al (2017) and Alqutaibi (2020) who showed that patient satisfaction is higher among a cohort of patients who received attachment‐retained RPDs. Attachment‐retained RPDs offer simplicity, effective resiliency, and stability, and reduce the torque on the abutment teeth, and most probably due to these features increase ME performance more than clasp‐retained RPDs (Alqutaibi 2020).

There were a few limitations to this study. First, this study was not designed as a randomized study and the patient's follow‐up was relatively short. Some other studies followed patients for several months after insertion of RPDs (Wallace et al. 2018; Fan et al. 2009). Second, the sample size of patients with triangular and quadrangular denture support of RPD was relatively small and results might be impaired. Also, only ME was measured as an outcome, and patient satisfaction questions were not included in the analysis. Yet many other studies excluded ME, and focused more on the diversity of possible tooth combinations, toothless areas, and prosthesis designs. Contrariwise, some researchers proposed that when analyzing teeth occlusions and insertion of RPDs in the studies, regardless of classification, it is important to analyze ME (Bae et al. 2017). Nevertheless, prosthetic rehabilitation with RPDs and evaluation of ME provide benefits and therefore is a measurement of patients' satisfaction. Consequently, additional studies with a larger sample size and longer follow‐up period are more prone to accurately evaluate the ME in patients with RPDs (Fuentes et al. 2018).

## 5 Conclusion

The insertion of RPDs significantly enhances ME, with this effect becoming particularly pronounced 3 months post‐insertion. Attachment‐retained RPDs were found to offer superior masticatory support and efficiency compared to clasp‐retained alternatives, highlighting the critical influence of precise prosthetic rehabilitation and RPD design on clinical outcomes. Moreover, our GLMM analysis revealed the significant impact of time on ME (*F*(3, 511) = 4.926, *p* = 0.002), demonstrating a universal improvement in masticatory function over time that was not significantly influenced by the type of RPD, the support type, or their interactions. This suggests that the choice of denture design affects not only immediate functionality and patient adaptation but also has a pivotal role in the long‐term success of the treatment, contributing to improved ME across all RPD designs. These results underscore the importance of considering both the type of retention and the design of RPDs in optimizing prosthetic rehabilitation to enhance patient satisfaction and treatment efficacy, while also indicating that the temporal aspects of treatment can lead to universal improvements in masticatory function, irrespective of the specific RPD design employed.

## Author Contributions

Conceptualization: Linda J. Dula, Kujtim Sh. Shala, David Stubljar, and Andrej Starc. Data curation: Linda J. Dula and Kujtim Sh. Shala. Formal analysis: Linda J. Dula, David Stubljar, Andrej Starc, and Shera Kosumi. Investigation: Linda J. Dula and Kujtim Sh. Shala. Methodology: Linda J. Dula and Kujtim Sh. Shala. Supervision: Kujtim Sh. Shala and David Stubljar. Validation: David Stubljar and Andrej Starc. Roles/writing – original draft: Linda J. Dula and Kujtim Sh. Shala. Writing – review and editing: Linda J. Dula, Kujtim Sh. Shala, David Stubljar, and Andrej Starc.

## Conflicts of Interest

The authors declare no conflicts of interest.

## Data Availability

The data that support the findings of this study are available from the corresponding author upon reasonable request.
